# De-Escalating Treatment Strategies for Patients with Human Epidermal Growth Factor Receptor-2 (HER2)-Positive Early-Stage Breast Cancer

**DOI:** 10.3390/cancers16203478

**Published:** 2024-10-14

**Authors:** Hikmat Abdel-Razeq

**Affiliations:** 1 Section of Hematology and Medical Oncology, Department of Internal Medicine, King Hussein Cancer Center, Amman 11941, Jordan; habdelrazeq@khcc.jo; Tel.: +962-6-5300460 (ext. 1000); 2 School of Medicine, University of Jordan, Amman 11941, Jordan

**Keywords:** breast cancer, HER2, trastuzumab, pertuzumab, de-escalation, personalized medicine, targeted therapies, adjuvant therapies, neoadjuvant therapies

## Abstract

**Simple Summary:**

Almost one in five patients with breast cancer have an aggressive subtype that expresses Human Epidermal Growth Factor-2 (HER2) receptor. The introduction of anti-HER2 therapy, like trastuzumab and pertuzumab, has dramatically improved treatment outcomes. However, such therapy is lengthy, costly, and can result in substantial cardiac toxicities. In this review, we discuss ways to de-escalate anti-HER2 therapy by shortening the treatment course to less than the 12-month standard, or minimizing companion chemotherapy. New molecular tools are emerging that should help physicians select which patients with HER2-positive breast cancer benefit most from aggressive and lengthy treatment regimens utilizing single or dual anti-HER2 therapy alone or in combination with chemotherapy.

**Abstract:**

Almost one-fifth of breast cancer cases express Human Epidermal Growth Factor-2 (HER2), and such expression is associated with highly proliferative tumors and poor prognosis. The introduction of anti-HER2 therapies has dramatically changed the natural course of this aggressive subtype of breast cancer. However, anti-HER2 therapy can be associated with substantial toxicities, mostly cardiac, and high cost. Over the past few years, there has been growing interest in de-escalation of anti-HER2 therapies to minimize adverse events and healthcare costs, while maintaining the efficacy of treatment. Data from clinical observations and single-arm studies have eluted to the minimal impact of anti-HER2 therapy in low-risk patients, like those with node-negative and small tumors. Though single-arm, the APT trial, in which patients with node-negative, small tumors received single-agent paclitaxel for 12 cycles plus trastuzumab for 1 year, was a practice-changing study. Several other recently published studies, like the PERSEPHONE trial, have shown more convincing data that 6 months of trastuzumab is not inferior to 12 months, in terms of disease-free survival (DFS), suggesting that de-escalating strategies with shorter treatment may be appropriate for some low-risk patients. Other de-escalating strategies involved an adaptive, response-directed approach, and personalized therapy that depends on tumor genomic profiling.

## 1. Introduction

Breast cancer continues to be the most diagnosed cancer among women worldwide [1,2]. Almost one in five women with breast cancer has human epidermal growth factor receptor-2 (HER2)-positive disease, which means over 450,000 cases are diagnosed every year worldwide [3,4,5]. HER2-positive breast cancer is known for its aggressive clinicopathological features and poor prognosis [6,7]. However, this aggressive behavior can be offset by the utilization of anti-HER2 targeted therapy. In one of the earliest clinical trials which examined the clinical impact of anti-HER2 therapy, Slamon et al. had shown that the prognosis of patients with HER2-positive metastatic breast cancer, when treated with trastuzumab, become similar to those with HER2-negative disease [8]. In early-stage breast cancer (EBC), several major clinical trials and meta-analyses had established the great benefit of trastuzumab and pertuzumab when combined with chemotherapy, both in the adjuvant and neoadjuvant settings [9,10,11,12,13]. However, there is disagreement on aggressiveness of therapy in a subgroup of HER2-positive patients with low-risk features. This group includes those with node-negative disease and small tumors, more so with tumors sized 10 mm or smaller (T1a-T1b). The management of such patients is highly variable across physicians and institutions [14,15,16]. In a recent study that investigated the variations in clinical management of patients with node-negative small tumors, investigators utilized an online questionnaire conducted across 70 breast medical oncologists in Spain. The questionnaire included 37 questions regarding management decisions of HER2-positive early breast cancer. Oncologists’ responses were very heterogenous; 53% would recommend upfront surgery, thus avoiding neoadjuvant therapy for node-negative tumors measuring 1.0 cm or less. When asked about de-escalating the duration of anti-HER2 therapy for small tumors, 56% and 69% of responders were open to de-escalate the duration of adjuvant trastuzumab in pT1bN0 and pT1aN0 tumors, respectively [17]. Clinicopathological features, like young age, negative estrogen receptors (ERs), high grade, and high Ki-67 may influence the aggressiveness of suggested treatment for patients with node-negative small tumors [17]. In this review, we will discuss the rationale for de-escalation first, then address de-escalation strategies.

## 2. Rationale for De-Escalation

De-escalation refers to strategies that intend to reduce the duration, intensity, or complexity of the anti-HER2 treatment or the companion chemotherapy without compromising treatment outcomes [18]. Emerging data suggest that for certain low-risk patients, less intensive regimens, including both the anti-HER2 agents and the chemotherapy, may achieve comparable outcomes to standard aggressive protocols. Identifying these subsets through biomarkers and clinical characteristics is crucial for effective de-escalation.

### 2.1. Toxicity Reduction

Anti-HER2 therapies, particularly when combined with chemotherapy, can cause significant adverse effects, most importantly cardiotoxicity, but also including gastrointestinal and hematologic toxicities. De-escalation may thus improve the quality of life of such patients.

#### 2.1.1. Cardiac Toxicity

Patients with breast cancer are at higher risk for cardiac adverse events secondary to chemotherapy, mostly anthracyclines and anti-HER2 therapy [19,20]. Radiation therapy, especially when it involves left-sided tumors, may add to this risk. In a recently published study, 829 patients with breast cancer (median age at diagnosis 54.2 years) who completed chemotherapy, including cardiotoxic agents, underwent echocardiographic screening every 2 years. Cardiac dysfunction was defined as left ventricular ejection fraction (LVEF) <50% after therapy initiation and included early- and late-onset cardiac dysfunction. Both anthracyclines and anti-HER2 (trastuzumab/pertuzumab) were given to 6.2% of the patients, while 16% received trastuzumab/pertuzumab alone, 39.7% received anthracyclines alone, and 38.1% received radiation alone. At a median follow-up of 8.6 (range, 1.8–39.8) years, and a total of 2,808 echocardiograms performed, the cumulative incidence of cardiac dysfunction increased from 1.8% at 2 years to 15.3% at 15 years from therapy initiation. In multivariable analysis, anthracyclines and trastuzumab/pertuzumab [hazard ratio (HR), 3.92, 95% CI, 1.74–8.85], anthracyclines [HR, 2.35 (95% CI, 1.25–4.4)], and non-Hispanic Black race [HR, 2.15 (95% CI, 1.37–3.38)] were important determinants of cardiac toxicity. Early-onset cardiac dysfunction was most prevalent among patients exposed to the combination of anthracyclines and trastuzumab/pertuzumab, while late-onset cardiac dysfunction was most prevalent among anthracycline- and radiation-exposed patients [21]. Such findings provide evidence to support the need for echocardiographic surveillance for several years after treatment with cardiotoxic agents and suggest a need to optimize cardiovascular risk factors to mitigate this potentially serious adverse event. Additionally, potential cardiac dysfunction can be a rationale to de-escalate anti-cancer therapy, when possible.

#### 2.1.2. Hematological and Gastrointestinal Toxicities

Toxicities of anti-HER2 therapy, beyond the cardiac ones, are encountered significantly more when trastuzumab is combined with pertuzumab. Diarrhea, alopecia, and nausea are relatively common. However, leukopenia, neutropenia, and febrile neutropenia were seen in patients receiving concurrent chemotherapy, which is likely the cause of most of these toxicities.

### 2.2. Cost-Effectiveness

Anti-HER2 therapy is lengthy and expensive, and the financial burden on patients themselves and healthcare systems can be substantial [22,23,24]. Obviously, de-escalation can lead to significant cost savings. In a study aimed to systematically review economic evaluation (EE) of adjuvant trastuzumab compared with chemotherapy alone for HER2-positive EBC, authors included 22 eligible studies from high-income (HICs) and upper-middle income countries (UMICs). Incremental cost-effectiveness ratios (ICERs) were within the cost-effectiveness thresholds of HICs, but not UMICs [25]. Several other studies, from low-income countries (LICs) reached a conclusion that one year of adjuvant trastuzumab therapy for HER2-positive EBC, when compared to chemotherapy alone, may not represent value for money in such countries [26,27,28,29]. Obviously, things can be even worse when dual anti-HER2 therapy, both trastuzumab and pertuzumab, is used in the adjuvant or neoadjuvant therapy [30,31]. The introduction of generic drugs and biosimilars, if priced at a significantly discounted rate, especially for resource-restricted countries, should improve patients’ access to such drugs [32,33].

## 3. Approaches to De-Escalation

Several strategies have been explored to de-escalate the therapy for patients with HER2-positive disease, including the anti-HER2 therapy itself, the companion chemotherapy, or both. This de-escalation can range from reducing the duration of therapy to minimizing the use of concurrent chemotherapy. While de-escalation strategies in high-income countries may be driven by factors related to toxicities, quality of life, and cost-effectiveness, cost and availability of anti-HER2 drugs, especially in low-income countries, may dictate de-escalation strategies. The lack of special funds to cover anti-HER2 therapy for refugees hosted in resource-restricted countries forced oncologists to delete anti-HER2 drugs, even for high-risk patients [23]. In a study conducted by our group, treatment outcomes of 113 Syrian refugees with breast cancer were reviewed. Though the majority of the patients received systemic chemotherapy, when indicated, only 11 (35.5%) of 31 patients with HER2-positive disease received any anti-HER2 therapy. Across all needed treatments, 37 (32.7%) patients had considerable deviations when judged against our institutional clinical practice guidelines (CPGs). Both DFS and OS of patients involved were significantly lower than patients treated at the same institution with no deviation [23].

### 3.1. Shortening the Duration of Trastuzumab

Since its introduction, and based on many clinical trials, the standard duration of trastuzumab therapy has been 12 months [9]. However, several trials have attempted to shorten this duration to 6 months or shorter (Table 1).

#### 3.1.1. Longer Is Not Necessarily Better

The landmark study, the HERA (HERceptin Adjuvant) trial, had a third arm which compared 2 years vs. 1 year of trastuzumab therapy after standard adjuvant chemotherapy, neoadjuvant chemotherapy, or both in 5102 patients with HER2-positive EBC [9]. After a median follow-up of 8 years, 2 years of adjuvant trastuzumab was not more effective than one year of treatment (HR 0·99; 95% CI, 0.85–1.14, *p* = 0.86). However, grade 3–4 adverse events and decrease in LVEF during treatment were reported more frequently in the 2-year treatment group (20.4% and 7.2%) than in the 1-year group (16.3% and 4.1%), respectively [34].

#### 3.1.2. Ultrashort Trastuzumab, the 9-Week Attempt

##### The FINHER Study

The FINHER trial was among the very first studies that attempted to shorten the duration of anti-HER2 therapy. However, the chemotherapy regimen used is not among the known standard ones now or back then. In this study, 1010 patients with node-positive or high-risk node-negative breast cancer were randomly assigned to receive three cycles of docetaxel or vinorelbine, followed in both groups by three cycles of FEC (fluorouracil, epirubicin, and cyclophosphamide). Women with HER2-positive disease (n = 232) were assigned to receive or not receive trastuzumab for 9 weeks, along with docetaxel or vinorelbine. After a median follow-up of 62 months, patients treated with trastuzumab tended to have better distant disease-free survival (dDFS) than those treated with chemotherapy only (HR 0.65; 95% CI, 0.38–1.12; *p* = 0.12). The median LVEF of trastuzumab-treated patients remained unaltered during the 5-year follow-up; however, only one woman treated with trastuzumab was diagnosed with heart failure [35]. Though this trial did not compare long vs. shortened trastuzumab therapy, it did pave the way for more studies to address this question. Additionally, patients included were high-risk (by inclusion criteria); the de-escalation strategies might not be their best option, if they can tolerate such therapy.

##### The SOLD Trial

The SOLD trial, an open-label randomized clinical trial, was closer to the standard of care in its choice of chemotherapy. A total of 2176 patients with HER2-positive EBC were randomized into two groups and the chemotherapy regimen was identical in the two groups: three cycles of 3-weekly docetaxel plus trastuzumab for 9 weeks, followed by three cycles of FEC. The first group (short trastuzumab) received no further trastuzumab, while the other group continued the drug for one full year from the start date. The study was updated recently, at a median follow-up of 8.1 years, and non-inferiority of the 9-week trastuzumab treatment could not be demonstrated for disease-free survival (DFS). However, the 5-year and 10-year OS rates were comparable between the 9-week (95.0% and 89.1%) and 1-year groups (95.9% and 88.2%); HR for all time points, 1.20; 90% CI, 0.94–1.54). Four patients (0.2%) died of a cardiac cause; three (75.0%) of these patients received trastuzumab for 9 weeks [36,37]. Failure to achieve its target may be a reflection of patients included in the study. To be realistic, high-risk HER2-positive patients may not be the best candidates for de-escalation trials. Many of such patients were enrolled in the SOLD trial, including 34% with ER-negative disease, 12% with stage III, and 11% with 4+ axillary lymph nodes.

##### The ShortHER Trial

This is the third study attempting the shorter 9-week trastuzumab therapy, and was updated and published recently. The ShortHER trial was a phase 3, non-inferiority, randomized trial comparing 9 weeks vs. 12 months of adjuvant trastuzumab with chemotherapy in patients with HER2-positive EBC. Patients were randomized to anthracycline-taxane combination chemotherapy plus 12 months or 9 weeks of trastuzumab. At a median follow-up of 9 years, the 10-year DFS for the whole group was similar: 77% vs. 78% in the long vs. short trastuzumab arm, respectively. Ten-year OS was also similar: 89% vs. 88% in the long vs. short arm, respectively. However, findings were not the same when higher-risk patients with ≥4 involved axillary lymph nodes (N4+) were considered. In this high-risk subgroup, the 10-year DFS rates in the long vs. short arm were 63% vs. 53%, and the 10-year OS rates in long vs. short arm were 84% vs. 64%. The updated analysis of the ShortHER trial showed that 12-month trastuzumab remains the standard treatment for patients with high-risk (N4+) disease. However, numerically, the differences for the patients at low (N0) or intermediate risk (N1-3) are negligible [38].

#### 3.1.3. Six Months vs. 12 Months Trastuzumab

##### The PHARE Trial

Given the failure of the 9-week regimen, it was natural to try a longer course of trastuzumab. The PHARE trial was an open-label, randomized, phase 3 trial in many centers in France. Patients with HER2-positive EBC who had breast-axillary surgery and had been treated with at least four cycles of chemotherapy and up to 6 months of trastuzumab were randomized to continue trastuzumab for another 6 months (12 months total duration; control group) or to discontinue trastuzumab at 6 months (6 months total duration; experimental group). A total of 1691 patients were randomized to receive 12 months of trastuzumab and 1693 to receive 6 months of trastuzumab. After a median follow-up of 42.5 months, the 2-year DFS was 93.8% in the 12-month group and 91.1% in the 6-month group (HR 1.28; 95% CI, 1.05–1.56; *p* = 0.29). Cardiac events were reported significantly more in patients treated with the 12-month trastuzumab than those in the 6-month group; 5.7% vs. 1.9%, *p* < 0.0001 [39]. The study was updated few years later; at a median follow-up of 7·5 years, the authors concluded that shorter duration of anti-HER2 is not non-inferior to the standard 12-month regimen [40]. Similar to previously discussed studies, the PHARE trial also enrolled higher-risk patients, including 15.1% with 4+ axillary lymph nodes and 38.5% with HR-negative disease.

##### PERSEPHONE Trial

This phase 3 trial compared 6 months vs. 12 months of adjuvant trastuzumab in HER2-positive early breast cancer. It demonstrated non-inferiority of the shorter regimen in terms of DFS, suggesting that 6 months could be a viable option for many patients. In this open-label, randomized, phase 3 non-inferiority trial, patients with HER2-positive EBC were recruited from 152 centers in the UK. Patients had to have a clear indication for chemotherapy and were randomized to receive either 6-month or 12-month trastuzumab every 3 weeks, intravenously or subcutaneously, given in combination with chemotherapy. At a median follow-up of 5·4 years, DFS events occurred in 13% of 2044 patients in the 6-month group and in 12% of 2045 patients in the 12-month group. Four-year DFS was 89·4% in the 6-month group and 89·8% in the 12-month group (HR 1.07; 90% CI, 0.93–1.24), non-inferiority *p* = 0.011. Additionally, the 6-month trastuzumab treatment was associated with fewer severe adverse events (19%) compared to 24% in the 12-month group, *p* = 0·0002. Additionally, fewer patients in the 6-month group stopped the drug early because of cardiotoxicity, 3% compared to 8%, *p* < 0·0001 [41].

#### 3.1.4. The Meta-Analysis

Controversy continues regarding the optimal duration of trastuzumab, especially so after the encouraging results of the PERSEPHONE trial. This meta-analysis was performed to reassess the efficacy and safety of shorter durations of trastuzumab. A total of 11,496 patients who were enrolled in six studies were eligible. Disease-free survival was significantly improved with the 12-month trastuzumab regimen compared to shorter ones (HR = 1.13; 95% CI 1.03–1.25; *p* = 0.01). Similarly, OS was significantly better (HR = 1.16; 95% CI 1.01–1.32; *p* = 0.03). Survival benefits were more pronounced in patients with ER-negative and node-positive disease. However, patients treated with shorter duration experienced significantly fewer cardiac events (OR 0.52; 95% CI 0.43–0.62; *p* < 0.00001) [42].

Given the above data, and despite the well documented increased risk of cardiotoxicity, 12 months of adjuvant trastuzumab treatment offer a considerable survival advantage and should continue to be the standard and preferred treatment for HER2-positive EBC. However, shorter durations of trastuzumab therapy can be considered for patients with cardiac disease, those with small tumors, and with node-negative disease, especially in resource-restricted countries.

### 3.2. De-Escalating Concurrent Chemotherapy

Concurrent chemotherapy, with anti-HER2 therapy adds to toxicity, inconvenience, and cost of therapy. Several trials have evaluated reduced-intensity chemotherapy regimens with anti-HER2 in patients with low-risk HER2-positive EBC (Table 2).

#### 3.2.1. The APT Trial

The APT (Adjuvant Paclitaxel and Trastuzumab) was designed to address de-escalating chemotherapy in patients with small, node-negative, HER2-positive EBC. In this phase 2 study, 410 patients with HER2-positive, node-negative, small breast cancer with tumors 3 cm or smaller were treated with weekly paclitaxel and trastuzumab for 12 weeks, followed by trastuzumab alone for 9 more months, to finish a total of 12 months of therapy. The primary analysis demonstrated a 3-year DFS of 98.7% [42]. In a follow-up analysis with a median follow-up of 6.5 years, the 7-year DFS was 93% with only four (1.0%) distant recurrences, 7-year recurrence-free interval (RFI) was 97.5%, and the 7-year OS was 95% [43]. On further follow-up, the 10-year invasive disease-free survival (iDFS) was 91.3% (95% CI, 88·3–94·4), 10-year RFI was 96.3% (95% CI, 94.3–98.3), and 10-year overall survival was 94.3% (95% CI, 91.8–96.8), and 10-year breast cancer-specific survival (BCSS) was 98.8% (95% CI, 97.6–100.0) (Table 3) [45].

#### 3.2.2. The ATEMPT Trial

The study was designed to compare the incidence of clinically relevant toxicities (CRTs) in patients treated with ado-trastuzumab emtansine (T-DM1) vs. paclitaxel-trastuzumab (TH) and to evaluate iDFS in patients receiving T-DM1. Patients with stage I, HER2-positive EBC (n = 497) were randomly assigned 3:1 to T-DM1 (n = 383) or TH (n = 114). T-DM1 was given every 3 weeks for 17 cycles (total one year), while TH was given weekly for 12 weeks, followed by trastuzumab alone every 3 weeks for 39 weeks (total, one year). There was no difference in CRT in both groups; 46% of patients on T-DM1 and 47% of patients on TH. At its initial publication in 2021, T-DM1 was associated with excellent results; the 3-year iDFS was 97.8% [46]. The trial was updated in June 2024; after a median follow-up of 5.8 years, the 5-year iDFS was 97.0%, the RFI was 98.3%, the OS was 97.8%, and the BCSS was 99.4%. Though the study was not powered to study the difference between TH and T-DM1, the 5-year iDFS in the TH arm was 91.1% [47].

#### 3.2.3. ADAPT Trial

Several clinical trials have tried to plan and direct a personalized therapy for HER2-postive breast cancer patients based on their initial response. The ADAPT trial is a prospective, phase 2 study investigating the potential of personalized treatment based on early response to neoadjuvant therapy. Initial results indicate that patients who achieve pCR after short-term preoperative therapy might be candidates for de-escalated post-operative treatment. Patients (n = 134) were randomized to 12 weeks of trastuzumab and pertuzumab with or without weekly paclitaxel. Early response was defined as a decline in Ki-67 from baseline by 30% or more, or low cellularity (<500 invasive tumor cells) at biopsy performed 3 weeks after starting therapy. The pCR rate in the taxanes and dual blockade was unexpectedly high at 90.5%, compared to 36.3% in the non-chemotherapy arm. Among the trastuzumab/pertuzumab arm, 24/92 (26.1%) were classified as non-responders, and only 8.3% achieved pCR compared with 44.7% in responders (38/92). The study concluded that early non-responders treated with dual anti-HER2 therapy, without chemotherapy, strongly predict failure to achieve pCR [49]. In a follow-up analysis, authors found that omission of further chemotherapy had no negative impact on iDFS in patients with pCR and concluded that weekly paclitaxel plus dual HER2 blockade for 12 weeks can be a de-escalated neoadjuvant regimen in patients with HR-negative, HER2-positive EBC [50].

### 3.3. Monotherapy with Anti-HER2 Agents

For patients with small, low-risk tumors or those with contraindications to chemotherapy, anti-HER2 monotherapy might be an option. Studies have shown that trastuzumab monotherapy can be effective in specific patient populations, though it is generally less effective than combination therapy.

#### RESPECT Trial

In one open-label, randomized controlled study, 275 patients aged 70–80 (mean age, 73.5) years with surgically treated HER2-positive patients with EBC received trastuzumab alone or trastuzumab plus chemotherapy. The study was designed to see if trastuzumab alone is not inferior to trastuzumab plus chemotherapy. After a mean follow-up of 4.1 years, the 3-year DFS was 89.5% with trastuzumab monotherapy vs. 93.8% with trastuzumab plus chemotherapy (HR 1.36; 95% CI, 0.72–2.58; *p* = 0.51). So, the study failed to show that trastuzumab monotherapy is not inferior; however, the observed loss of survival without chemotherapy [restricted mean survival time (RMST)] differed by only −0.39 months at 3 years. Adverse events were more common with the combination arm and that translated into more deterioration in health-related quality of life (HRQoL) at 2 months (31% for trastuzumab monotherapy vs. 48% for trastuzumab and chemotherapy; *p* = 0.016), and at 1 year (19% vs. 38%; *p* = 0.009). Though the non-inferiority for trastuzumab monotherapy was not met, given the added toxicity, poor quality of life, and the little observed loss of survival without the addition of chemotherapy (less than 1 month at 3 years), trastuzumab monotherapy can be considered an alternative adjuvant therapy option for selected older patients like those enrolled in the RESPECT trial [48].

Targeted therapies such as tucatinib, trastuzumab deruxtecan, and neratinib have demonstrated impressive responses in advanced-stage breast cancer, including those with brain metastasis. Incorporating these agents, alone or in combination, in the post-neoadjuvant therapy may improve the prognosis of HER2-positive EBC [51]. The identification of biomarkers that help predict response to such agents may advance de-escalating strategies.

## 4. Biomarker-Guided Therapy

### 4.1. Image-Guided Therapy (PHERGain Trial)

As a continuation of the personalized approach, the PHERGain trial was conducted to optimize patient selection for anti-HER2 therapy utilizing positron emission tomography (PET) scans to identify patients who are likely to benefit from de-escalated neoadjuvant treatment. A total of 356 patients with HR-positive and HER2-positive breast cancer were randomized to two cycles of conventional TCHP regimen (docetaxel, carboplatin, trastuzumab, and pertuzumab) vs. a chemotherapy-free regimen with trastuzumab and pertuzumab in combination with endocrine treatment. Early metabolic response was evaluated by FDG-PET at baseline and after two cycles. Patients in the standard arm continued to receive TCHP for four more cycles. In the experimental arm, early responders continued to receive six more cycles of chemo-free treatment, while the non-responders were switched to receive six courses of TCHP. Following the eight cycles of the neoadjuvant chemotherapy-free group, a total of 38% of early responders achieved pCR and had a 3-year iDFS of 98.8%. However, when taken together, patients in the experimental arm had lower iDFS (95.4%) compared to the standard arm (98.3%). More outcome results, including OS, are still eagerly awaited [52,53].

Biomarkers that help predict response to anti-HER2 therapies can enable more personalized treatment approaches and thus de-escalation. The HER2DX risk score and tumor-infiltrating lymphocytes (TILs) are being studied to tailor therapy intensity based on individual risk profiles [54,55,56,57].

### 4.2. HER2DX Risk Score

Researchers from Spain and United States attempted to develop and validate a new risk scoring system (HER2DX) that can help oncologists decide on treatment aggressiveness of breast cancer patients with HER2-positive disease. The scoring system employs both clinical and genomic data to predict treatment response (pCR) and survival in early-stage HER2-positive breast cancer based on a 27-gene expression plus clinical features, tumor size, and nodal staging. The genomic data analysis utilizes four gene expression signatures tracking tumor cell proliferation, immune infiltration, luminal differentiation, and the expression of the HER2 amplicon. Various data sets, mostly based on the ShortHER database, were used to train, then verify and validate the predictive model. The HER2DX risk score was significantly associated with DFS in the ShortHER database (*p* = 0.002), and in an independent combined validation database; the 5-year DFS in the low-risk group was 97.4% compared to 84.7% in the high-risk group, *p* = 0.005. Overall survival was also better in the low-risk group (5-year OS: 95.8%) compared to 93.1% in the high-risk group, *p* = 0.016. Additionally, continuous HER2DX pCR likelihood score was significantly associated with pCR, *p* < 0.0001 [58].

In another independent study designed to test the ability of the HER2DX assay to predict the likelihood of pCR in patients with early-stage HER2-positive breast cancer who are receiving a de-escalated neoadjuvant therapy, pretreatment tumor biopsy samples from 80 of 97 patients enrolled in the single-arm, multicenter, prospective phase 2 DAPHNe clinical trial were used for HER2DX assay. Patients had newly diagnosed stage II-III HER2-positive disease and were treated with neoadjuvant paclitaxel weekly for 12 weeks plus trastuzumab and pertuzumab every 3 weeks for four cycles. The HER2DX pCR score was significantly associated with pCR; the pCR rates were 92.6% in the HER2DX high, 63.6% in the medium, and 29.0% in the low pCR score groups (high vs. low odds ratio, 30.6; *p* < 0.001). The researchers concluded that the HER2DX pCR score assay may predict pCR following treatment with de-escalated neoadjuvant paclitaxel with trastuzumab and pertuzumab in patients with early-stage HER2-positive disease, and as such, the HER2DX pCR score might guide management decisions by identifying patients who are candidates for de-escalated approaches [59]. Several other studies reached similar conclusions [60,61,62].

## 5. Future Directions and Conclusions

As our understanding of HER2-positive breast cancer biology advances, future research will hopefully refine de-escalation strategies further. Integration of tumor genomic profiling may help identify patients who might benefit from less intensive therapy. Future research should focus on exploring the molecular heterogeneity of HER2-positive breast cancer to identify new prognostic and predictive biomarkers which could pave the way toward the development of truly personalized less burdensome treatment options. Collecting and analyzing data from routine clinical practice (real-world data) to validate de-escalation approaches, away from the very stringent clinical trial setup, should help move such approaches faster. Additionally, exploring novel combinations of targeted therapies may help avoid or reduce the need for traditional toxic chemotherapy. It is hoped that the application of artificial intelligence (AI) should identify clinical, pathological, and molecular markers that may help oncologists decide on aggressiveness of anti-cancer therapy. It is important to emphasize here that de-escalating strategies addressed here might not be an option for higher-risk HER2-positive patients like those with four or more positive lymph nodes.

In conclusion, treatment de-escalation of HER2-positive breast cancer patients holds promise for reducing both toxicity and costs, while maintaining efficacy and outcomes. We believe that the current available evidence justifies some of the de-escalation strategies addressed in this review, especially in resource-restricted countries.

## Figures and Tables

**Table 1:** Duration of anti-HER2 therapy, short vs. long.

Variables	Study [References]	Publication Dates	Key Findings
One Year vs. 2 Years	HERA trial [34]	2005 and 2013	Two years is not better than one year (HR 0.99; 95% CI, 0.85–1.14, *p* = 0.86). Two years was associated with more grade 3–4 adverse events and decrease in LVEF.
Nine Weeks vs. 12 Months	FINHER [35]	2008	Nine weeks of trastuzumab tended to have better dDFS than chemotherapy only (HR 0.65; 95% CI, 0.38–1.12; *p* = 0.12). LVEF remained unaltered.
	SOLD trial [36,37]	2018, 2024	Nine weeks is not non-inferior to 12 months for DFS. No substantial difference in dDFS and OS between the short and long group.
	ShortHER [38]	2023	Nine weeks is not non-inferior to 12 months (in higher-risk patients with N4+).
Six Months vs. 12 Months	PHARE Trial [39,40]	2013 and 2019	Six months is not non-inferior to 12 months. Cardiac events: 5.7% (12-month), 1.9% (6-month), *p* < 0.0001.
	PERSEPHONE Trial [41]	2019	Six months is not inferior to 12 months; 4-year DFS 89.4% (6-month), 89.8% (12-month), HR 1.07, 90% CI 0.93–1.24, non-inferiority *p* = 0·011. Six-month treatment was associated with fewer severe adverse events.
Meta-analysis (Short vs. Long Duration)	Six studies (patients treated between 1999–2015) [42]	2019	DFS and OS were significantly improved with the 12-month trastuzumab regimen compared to shorter ones.

HER2: Human epidermal growth factor-2; HR: Hazard ratio: LVEF: Left ventricular ejection fraction; dDFS: Distant disease-free survival; DFS: Disease-free survival; OS: Overall survival; N4+: Four or more axillary lymph nodes involved.

**Table 2:** De-escalation of chemotherapy.

Strategy	Variables	Studies [References]	Publication Date(s)	Key Findings
De-escalating Concurrent Chemotherapy	Single-agent paclitaxel weekly for 12 weeks plus trastuzumab for 12 months	APT trial (Single-arm) [43,44,45]	2015, 2019, 2023	10-year iDFS: 91.3%. 10-year RFI: 96.3%. 10-year OS: 94.3%. 10-year BCSS: 98.8%.
	T-DM1 vs. paclitaxel (12 weeks) plus trastuzumab (12 months)	ATEMPT trial (Randomized) [46,47]	2021, 2024	Efficacy (T-DM1 arm): 5-year iDFS: 97.0%, 5-year RFI: 98.3%, 5-year OS: 97.8%, 5-year BCSS: 99.4%. Efficacy (TH arm): 5-year iDFS: 91.1%. Adverse events: No difference in CRT in both groups.
Monotherapy with anti-HER2 Agents (no chemotherapy)	Elderly patients (70–80 years); trastuzumab alone vs. trastuzumab plus chemotherapy	RESPECT (Open-label, randomized) [48]	2020	Trastuzumab monotherapy is not non-inferior to trastuzumab plus chemotherapy. Combination was associated with more AE and more deterioration in HRQoL.

iDFS: Invasive disease-free survival; RFI: Relapse-free interval; OS: Overall survival; BCSS: Breast cancer-specific survival; TH: Paclitaxel plus Trastuzumab; CRT: Clinically relevant toxicities; AE: Adverse events; HRQoL: Health-related quality of life.

**Table 3:** Survival outcomes of patients treated on the APT trial.

Outcome	At 3-Year (2015)	At 7-Year (2019)	At 10-Year (2023)
Invasive Disease-Free Survival (iDFS)	98.7 (95% CI, 97.6–99.8),	93% (95% CI, 90.4–96.2)	91.3% (95% CI, 88.3–94.4)
Recurrence-Free Interval (RFI)	99.2% (95% CI, 98.4–100.0)	97.5% (95% CI, 95.9–99.1)	96.3% (95% CI, 94.3–98.3)
Overall Survival (OS)	NR	95.0% (95% CI, 92.4–97.7)	94.3% (95% CI, 91·8–96·8)
Breast cancer-specific survival (BCSS)	NR	98.6% (95% CI, 97.0–100.0%)	98.8% (95% CI, 97.6–100.0)

iDFS: Invasive disease-free survival; RFI: Relapse-free interval; OS: Overall survival; BCSS: Breast cancer-specific survival; CI: Confidence interval; NR: Not reported.
